# Diagnostic and therapy gaps in BPPV care: a retrospective analysis and feasibility evaluation across a London NHS trust

**DOI:** 10.3389/fneur.2026.1950987

**Published:** 2026-10-14

**Authors:** Vassilios Tahtis, Abigail Beddard, Jessica Spall, Caroline Hare, James E. Scott, Lisa-Jane Fowler, Rebecca M. Smith, Yee Mah, Diego Kaski

**Affiliations:** 1 Kings College Hospital NHS Foundation Trust, London, United Kingdom; 2 Department of Brain Sciences, Charing Cross Hospital, Imperial College London, London, United Kingdom; 3 Department of Biomedical Engineering, School of Biomedical Engineering and Imagine Sciences, Kings College London, London, United Kingdom; 4 National Hospital for Neurology and Neurosurgery, Queen Square, London, United Kingdom

**Keywords:** benign paroxysmal positional vertigo (BPPV), dizziness, emergency department, falls, vertigo

## Abstract

**Background:**

Benign paroxysmal positional vertigo (BPPV) is the most prevalent cause of peripheral dizziness, yet it remains poorly assessed and treated in acute settings. Despite the availability of validated bedside tools such as the Dix-Hallpike test (DHT) and canal repositioning manoeuvres (CRM) such as the Epley, diagnostic variability is high, neuroimaging is overused, and vestibular suppressants are frequently prescribed. This two-part evaluation characterised BPPV assessment and management across a London NHS Trust and assessed the feasibility of incorporating DHT into an existing AHP-led falls pathway within the emergency department (ED).

**Methods:**

A two-part evaluation was conducted across a large London NHS teaching hospital. Part 1 comprised a retrospective review of electronic patient records to characterise current BPPV assessment and management practices and identify potential service gaps. Part 2 was a prospective feasibility study evaluating the incorporation of AHP-led DHT screening into routine falls assessments for patients aged ≥65 years presenting to the ED.

**Results:**

Part 1 identified 128 cases (mean age 63.4 years; 61.7% female). BPPV was the most common discharge diagnosis, 45.3% (95% CI 37.0–53.9%), yet full assessment and treatment were documented in only 14.8% of cases (95% CI 9.7–22.0%). Vestibular suppressants were prescribed in 43.8%, neuroimaging performed in 71.9 and 18.8% left without a definitive diagnosis. Readmission for dizziness within three months occurred in 13.3% (95% CI 8.5–20.2%). Part 2 included 102 patients, 79 with complete records (mean age 84.6 years; 54% female). DHT was completed in 62% (63/102 attempts; 95% CI 52.1–70.6%); BPPV was identified in 8% (95% CI 4.0–14.7%) and all received CRM. No dizziness-related readmissions occurred at three months.

**Conclusion:**

This evaluation identifies consistent gaps in acute BPPV assessment and management, including overuse of neuroimaging and vestibular suppressants alongside underutilisation of validated bedside tools. Physiotherapy led DHT screening is feasible in a busy ED setting, even in older adults with multiple comorbidities, and the absence of readmissions in this small feasibility cohort is an encouraging finding that warrants evaluation in a larger, controlled cohort.

## Introduction

Dizziness and vertigo are common reasons for hospital attendance, accounting for up to 13% of emergency department (ED) presentations (1), with over 20% resulting in admission (2). Because these symptoms span a broad pathological spectrum and significantly impact quality of life, timely accurate diagnosis is important (3). The majority of presentations are attributed to benign inner ear conditions, most commonly Benign Paroxysmal Positional Vertigo (BPPV) and acute unilateral peripheral vestibulopathy (vestibular neuritis, and labyrinthitis). Dizziness can also indicate serious pathology, such as stroke, requiring skilled assessment (4, 5).

BPPV is the most prevalent cause of peripheral dizziness worldwide, with an estimated lifetime prevalence of 2.4% (6). Caused by displaced otoconial debris within the semicircular canals, it is readily identifiable and treatable at the bedside using canal repositioning manoeuvres (CRM).

Despite its prevalence, BPPV remains poorly managed in acute settings. Many ED clinicians report limited training and familiarity with bedside assessment, leading to high diagnostic variability (7, 8). Nearly half of patients presenting with dizziness to the ED leave without a definitive diagnosis, increasing vestibular suppressant prescription, unnecessary neuroimaging, and mismanagement (9, 10). Misdiagnosed dizziness, particularly in older adults, is associated with reduced health-related quality of life and increased falls risk. As such current guidelines recommend routine BPPV screening for fallers regardless of symptoms (11–14).

The Dix-Hallpike Test (DHT) and the Epley CRM are well-established, quick, and effective bedside tools, yet both remain underutilised in the ED due to logistical barriers and training gaps (15). Allied health professionals (AHPs), including physiotherapists and occupational therapists have demonstrated they can improve guideline adherence and quality of care without extending length of stay or increasing adverse events (16–18).

Given the high prevalence of BPPV, its frequent misdiagnosis, and the established role of AHPs in vestibular care, this two-part evaluation was designed to first characterise current assessment and management of BPPV within acute care and identify potential gaps in practice. The second component evaluated whether BPPV screening using the DHT could be feasibly incorporated into an existing allied health professional-led falls pathway within the emergency department as a potential strategy to address these gaps. Together, the two components were intended to describe current care and explore a pragmatic approach to improving identification and management of BPPV.

Therefore, this evaluation had two linked objectives. The primary objective of Part 1 was to characterise assessment and management of patients in whom BPPV was diagnosed, assessed, or considered within acute care, and to identify potential gaps in practice. The primary objective of Part 2 was to evaluate the feasibility of implementing allied health professional-led DHT screening within a routine emergency department falls pathway. Secondary objectives included describing diagnostic yield, treatment delivery, implementation barriers, and short-term dizziness related reattendance.

## Materials and methods

### Study design

This evaluation comprised two linked components. First, a retrospective review of electronic patient records (EPR) was undertaken. Second, a prospective feasibility evaluation examined the incorporation of AHP led posterior canal (PC) BPPV screening using DHT into routine falls assessments for older adults in the emergency department. The completed STROBE checklist is provided as Supplementary Material. Part 1 drew on entries from both Trust sites, with data presented in aggregate rather than by centre, as the retrospective review characterised Trust-wide practice; Part 2 was conducted at a single ED site.

All patients received routine care at King’s College Hospital NHS Foundation Trust. This study was registered at the Trust as a service evaluation and data was handled in accordance with institutional policies. Data were pseudonymised prior to analysis, and no patient-identifiable information is used or reported in this manuscript. Both the training of physiotherapists in DHT/CRM and the subsequent screening implementation were delivered within existing physiotherapy scope of practice as a standard clinical skills and quality improvement initiative, rather than as a research intervention or a change to care outside normal practice.

### Statistical analysis

Data were summarised and analysed descriptively in Microsoft Excel. For selected key proportions, 95% confidence intervals (CI) were calculated using the Wilson method to provide estimates of precision. No formal hypothesis testing or between group inferential analyses were undertaken.

### Part 1: retrospective clinical notes evaluation

A retrospective review of EPR was conducted across two hospital sites within the Trust, using the CogStack application framework (19), a clinical data-processing platform enabling medical record search. Clinical entries spanning a consecutive seven-month period (1st June 2022 to 31st December 2022). Clinical entries generated as part of the Part 2 feasibility implementation were not a component of the Part 1 dataset.

A CogStack search was used to identify medical record entries containing the following predefined terms: BPPV, Labyrinthitis, Ménière’s, Vestibular, Dix Hallpike, Semont, and Epley. Terms were selected by consensus among the study team to capture records where BPPV was diagnosed, assessed, or explicitly considered. Broad symptom terms such as ‘dizziness’ or ‘unsteadiness’ were deliberately excluded to reduce false positives and limit review burden, as these commonly encompass heterogeneous, non-vestibular presentations. Manoeuvre-specific terms were included to identify cases with documented diagnostic and therapeutic assessment where diagnostic coding may have been incomplete. The query encompassed all instances across the EPR, not limited to ED entries.

The extracted dataset contained timestamped instances where search terms appeared, alongside excerpts of corresponding clinical entries. Duplicate, repeat, and non-contributory entries were reviewed and excluded by trained clinicians (VT, LJF, JES, CH, AB). To ensure methodological rigour, a subset of five cases was jointly reviewed by all raters. Discrepancies were resolved by consensus before proceeding to full dataset analysis. Five therapists independently reviewed the dataset and categorised each case using a predefined framework. The framework consisted of a structured data extraction proforma containing predefined categorical fields. A supporting guidance document provided operational definitions and classification rules for all extracted variables and outcome categories. To promote consistency, reviewers completed an initial calibration exercise using sample cases before undertaking independent review. Ambiguous cases were discussed at regular meetings and resolved by consensus.

The help sheet, blank categorisation proforma, and operational definitions for extracted variables and outcome classifications are provided as Supplementary Material.

### Part 2: real-world feasibility implementation

#### Screening procedure

A prospective feasibility evaluation was undertaken for one month (1st to 31st December 2022) at one ED site within the Trust, examining the incorporation of the DHT into routine falls assessments for patients aged ≥65 years. Screening aligned with the World Falls Guidelines (12) and local governance frameworks.

#### Physiotherapy training and implementation

Ten members of the ED physiotherapy team received structured training in PC BPPV assessment and management over a two-week period. Training focused on posterior canal BPPV only, as this is the most commonly affected canal (6). It was delivered by a principal expert trainer (JS) and comprised face-to-face sessions, online modules, and practical components. Regular reviews with the trainer were scheduled to address clinical uncertainties and ensure protocol adherence. Training included standard screening for contraindications to DHT/CRM, including restricted cervical range of movement, cervical spine instability, and vascular risk factors for vertebrobasilar insufficiency, consistent with established manoeuvre safety guidance. Assessment was not undertaken where clinical screening identified any such contraindications.

#### Assessment protocol

All eligible patients underwent a DHT on hospital beds or trolleys, with both left and right canals assessed. Where testing was positive, an immediate CRM was performed following informed consent. The affected canal was subsequently reassessed with the DHT to confirm resolution. Assessment was undertaken as part of routine clinical care; video recordings of eye movements were not obtained. Nystagmus was assessed through direct clinical observation by trained physiotherapists, who received specific training in vestibular assessment and management, including recognition of positional nystagmus during Dix-Hallpike testing, prior to participation. Video-oculography goggles were not used, consistent with standard bedside DHT practice in this setting.

#### Outcome metrics for feasibility study

Outcome metrics included: the number of positive BPPV assessments; instances where assessment or treatment could not be completed, with documentation of limiting factors; and fidelity of implementation, assessed by the principal expert trainer through review of physiotherapist documentation. Falls were classified according to intrinsic and extrinsic causal factors (20).

This study was registered as a service evaluation with King’s College Hospital NHS Trust (InPhase: #1270) and did not require NHS Research Ethics Committee approval according to UK HRA guidance. The evaluation was approved by the King’s College Hospital NHS Foundation Trust Therapy Governance Committee.

## Results

### Part 1: retrospective clinical notes evaluation

The initial query identified 1,140 document entries containing one or more search terms. Following removal of duplicates and non-contributory records, 193 unique patient cases were identified, of these 128 were included in the final analysis. A flow diagram summarising case identification and exclusion for both Part 1 and Part 2 is provided as Supplementary Figure 1. No imputation was performed for missing or incomplete data; a complete-case approach was used throughout and all figures represent descriptive statistics on the available data.

Table 1 provides detailed patient characteristics. The mean age of included patients was 63.4 years (SD 20), with females comprising 61.7% of the sample. Comorbidities were frequent, most notably cardiac or vascular risk factors (27.3%), anxiety or depression (14.8%), and previous stroke (10.2%).

**Table 1:** Patient characteristics of clinical notes evaluation (*n* = 128).

Variable	Results	%
Age years (SD)	63.4 (20)	
Sex		
Female	79	61.7
Male	49	38.3
Comorbidities		
Previous Stroke	13	10.2
Cardiac history/vascular risk factors	35	27.3
Migraine	9	7
Anxiety/depression	19	14.8
Presenting complaint		
Dizziness	66	51.6
Vertigo	12	9.4
Lightheadedness	2	1.6
Unsteadiness	4	3.1
Fall	14	10.9
Headache/migraine	4	3.1
Other	26	20.3
Acute Neuroradiology		
CT head	59	46.1
MRI head	6	4.7
CT and MRI head	26	20.3
Angiography CT/MRI	24	18.8
None	36	28.1
Medication on discharge		
Vestibular suppressants	56	43.8
Location of the care episode		
ED (emergency department)	90	70.3
Acute wards	38	29.7
Vestibular bedside assessment		
Only assessment manoeuvre completed (Dix-Hallpike)	17	13.3
Both assessment and treatment completed	19	14.8
Discharge diagnosis		
BPPV	58	45.3
Vestibular neuritis	4	3.1
Other peripheral	5	3.9
Migraine	3	2.3
Stroke	1	0.8
Other central cause	12	9.4
Other	21	16.4
Unknown	24	18.8
Outcome		
Discharged from ED	77	60.2
Admitted from ED	9	7
Already inpatient	42	32.8
Readmission within 3 months for dizziness related episode	17	13.3
Number of teams seen/specialist teams for same problem		
One team	79	61.7
Two teams	35	27.3
Three or more teams	14	10.9
Onward referral		
ENT/Specialist Vestibular service/audiology	15	11.7
Outpatient physiotherapy neurology clinic	11	8.6
GP	23	18
None	49	38.3
Not known	4	3.1
Cardiac services	1	0.8
Falls clinic	0	0
Outpatient neurology clinic	3	2.3
Stroke unit	3	2.3
Other	19	14.8
Initial health professional consultation		
Neurology specialist review	13	10.2
Non-neurology medical review	70	54.7
Nurse	6	4.7
Allied Health Professional	27	21.1
Vestibular Trained Allied Health Professional	7	5.5
Not specified	5	3.9

The predominant presenting complaint was dizziness (51.6%), followed by falls (10.9%) and vertigo (9.4%). Neuroimaging was common: with 71.9% of patients undergoing some form of acute neuroradiology. Vestibular suppressants were prescribed on discharge in 43.8% of cases. Over 13% of patients were readmitted within three months with a dizziness-related presentation as the primary reason.

Most care episodes occurred in the ED (70.3%), with 29.7% managed on acute wards. Dedicated vestibular assessment was documented in 28.9% overall, comprising any of: HINTS(+), gaze test/nystagmus, pursuit, head impulse test (HIT), test of skew, Romberg, DHT, or supine roll test. A total of 13.3% (95% CI 8.5–20.2%) underwent assessment manoeuvres only and 14.8% (95% CI 9.7–22.0%) had both assessment and treatment completed. Discharge diagnoses most frequently reflected peripheral vestibular causes, BPPV was the most common (45.3%; 95% CI 37.0–53.9%). Central causes accounted for 12.5, and 18.8% were recorded as unknown. Interestingly, in six cases (4.7%), the clinical presentation raised sufficient concern for possible stroke to warrant urgent assessment (suspected stroke was recorded under “other” in the Presenting Complaint section of Table 1, as it represents a working diagnosis rather than a discrete symptom). The single case recorded with a discharge diagnosis of stroke (Table 1, 0.8%) was a distinct diagnosis; no patient in either part of this evaluation received simultaneous diagnoses of BPPV and acute stroke.

### Part 2: real-world feasibility implementation

A total of 102 patients were initially seen within the feasibility study; however, as much of the evaluation relied on paper records, complete records could only be reliably recalled for 79 patients. The missing records arose during retrospective reconciliation of paper-based documentation and could not be reliably recovered, necessitating a complete-case analysis. No attempt was made to impute missing data; analyses were restricted to available records. Participant characteristics are reported for the 79 patients with complete records, while selected implementation outcomes are reported for the full cohort of 102 patients (Table 2). This process is summarised in Supplementary Figure 1.

**Table 2:** Patient characteristics of real-world implementation (*n* = 79).

Variable	Results	%
	*n* = 79	
Age, years, mean, SD	84.6 (7.7)	
Female	43	54%
Comorbidities		
Previous stroke	8	10%
Cardiac history/vascular risk factors	48	61%
Migraine	0	0%
Anxiety/depression	12	15%
Presenting issue (besides fall)		
Perfusion/vascular instability	10	13%
Metabolic derangement	4	5%
Infection/inflammation	5	6%
Other	2	3%
Medication on discharge		
Vestibular suppressants	0	0%
Reason for fall		
Intrinsic	53	67%
Extrinsic	26	33%
**Readmission within 3 months for dizziness related episode**	0	0%
	*n* = 102	
Vestibular testing		
Dix Hallpike manoeuvre completed	63*	62%
BPPV diagnosis	8*	8%
Repositioning manoeuvre completed	8*	8%
Outcome		
Discharged from ED	49*	48%
Admitted from ED	9*	9%
Already inpatient	15*	15%
Discharged from ward	11*	11%
Not known	18*	18%
Onward referral		
ENT/specialist vest service/audiology	0*	0%
Neuro OP PT	0*	0%
GP	0*	0%
None	54*	53%
Not known	18*	18%
cardiac services	0*	0%
falls clinic	11*	11%
op neurology	0*	0%
stroke unit	0*	0%
Other	19*	19%

*Rows marked with an asterisk use denominator = 102 (initial cohort). All other rows use denominator = 79.

The mean age of the cohort was 84.6 years (SD 7.7), with females accounting for 54%. Cardiac or vascular risk factors were present in 61%, previous stroke in 10%, and anxiety/depression in 15%. Intrinsic causes accounted for the majority of falls (53 cases, 67%), with loss of balance (44%) and dizziness (10%) the most frequently reported in this category. Extrinsic factors were predominantly attributed to tripping (22%) or situational hazards. Of the 102 initially identified, 48% of patients were discharged directly from the ED, while 11% were discharged from inpatient wards. No patients were readmitted within three months for dizziness-related complaints. Onward referral was limited: 53% had no documented onward referral, 11% were referred to falls clinics, and 19% to other services.

DHT was attempted across the full initial 102 cohort of patients and was completed in 63 patients (61.8%; 95% CI 52.1–70.6%). BPPV was identified in 8 patients (7.8%; 95% CI 4.0–14.7%), all of whom received a CRM. Of the 102 cases, 39 did not undergo DHT. Environmental barriers included insufficient space in the ED and limited availability of trolleys. Patient-specific limitations are detailed in Table 3. All assessments were undertaken by trained members of the ED physiotherapy team during their normal working hours; staff availability and time of day were not contributing factors to non-completion.

**Table 3:** Reasons for not having an assessment manoeuvre completed as part of the feasibility study.

Reason	Number of patients
Pain	6
No bed available	13
Confusion	8
Nausea	2
Unable to tolerate	6
Declined	2
Intoxicated	1
Drowsy	1
**Total**	39

Among patients presenting with additional issues to a fall, the perfusion/vascular instability category was dominated by postural hypotension, which accounted for all ten cases (13%). Metabolic derangements (including those related to alcohol and hyponatraemia) and infection/inflammation (such as UTI, urosepsis, and respiratory infections) were less frequent. Two cases involved structural or primary organ pathology.

## Discussion

Our evaluation across two NHS hospitals demonstrated limited routine ED assessment and treatment of BPPV. Whilst BPPV is an easily treatable condition, it remains poorly assessed and managed in acute settings, in part due to the challenge of differentiating it from central conditions such as stroke. Despite advances in structured bedside approaches (including HINTS+, GRACE-3, TiTrATE, and STANDING) (4, 21–23), developing pathways that safely identify and treat benign causes remains difficult. A study within another London teaching hospital (24) identified that the current approach to acute vertigo in ED required change, though significant barriers remained.

Our patient population was predominantly female and older, with frequent comorbidities. Over half presented with dizziness or vertigo, with most episodes occurring in ED. Fifty-eight patients were diagnosed with BPPV at discharge, reflecting its status as the most common cause of peripheral dizziness (6). Despite this, only 14.8% of patients were assessed and treated for BPPV, and 13.3% assessed but not treated. These figures, whilst higher than those reported by Kerber et al. (15) remain low given the diagnostic rate. The observation that assessment was completed without treatment in a notable proportion of cases warrants further investigation, as the additional time required to perform a CRM once a DHT has been performed is small. Lloyd et al. (25) concluded that adherence to clinical guidelines for DHT and CRM was poor across healthcare systems, attributable to time constraints, lack of specialist training, and negative experiences. In our feasibility implementation, structured training with an expert trainer, regular reviews, and access to digital resources appeared to support adherence, with reasons for non-completion largely attributable to patient compliance and environmental factors, namely a lack of space.

The frequent use of neuroimaging is notable. Nearly half of patients received CT head, and a further 20.3% underwent both CT and MRI, yet 45.3% were ultimately diagnosed with a peripheral cause. Whilst this likely reflects appropriate concern for serious pathology given the prevalence of vascular risk factors and diagnostic uncertainty, it also illustrates an over-reliance on imaging despite its limited sensitivity for posterior circulation pathology within the first 24–72 h (22, 26) and the lack of utility of CT head for vertigo syndromes (22). An additional contributing factor may be pathway driven processes, as patients referred via a stroke call often undergo early neuroimaging with a low threshold. Confirmed stroke was rare when dizziness occurred in isolation, and all six cases were managed appropriately through established acute pathways with a discharge rate of 0.8% in our cohort, consistent with previous reports (2). The wider use of validated bedside tests could support more targeted imaging decisions (4, 21–23).

Nearly one-fifth of patients (18.8%) were discharged with an unknown diagnosis, consistent with the limited use of bedside vestibular assessment observed (28.9%). A substantial proportion were discharged on vestibular suppressants (43.8%), including many of those ultimately diagnosed with BPPV. NICE Guideline NG249, recommends DHT for rotational vertigo and does not recommend pharmacotherapy (14). NG249 (published 2025) is cited here as the current best-practice reference framework against which our findings are discussed, rather than as the standard in force at the time of data collection in 2022.

There was marked variation in the professionals completing initial assessments. Over half were seen by non-neurology doctors and approximately one-fifth by AHP staff, with only 5.5% seen by vestibular-trained AHPs. This variability is likely to contribute to the low uptake of DHT and CRM and the frequent use of neuroimaging. No patients in the audit cohort were referred to a falls clinic, despite 10.9% presenting following a fall, contrasting with the 14% falls clinic referral rate achieved in the feasibility study and highlighting a gap in practice.

Referral rates to specialist vestibular services were low overall, with only 11.7% referred to ENT or specialist vestibular services. Given that BPPV is frequently recurrent, the absence of structured follow-up may contribute to the 13.3% readmission rate within three months observed in this cohort. Ozgirgin et al. (27) reported CRM effectiveness of 80% on first attempt and 92% with repeated manoeuvres, suggesting that improved uptake of assessment and treatment, combined with appropriate onward referral, could meaningfully reduce readmission rates and the associated healthcare burden.

By contrast, the feasibility study cohort represents a distinct population from the broader evaluation undertaken in Part 1, and findings must be interpreted contextually rather than compared directly. The feasibility study adopted a pragmatic approach in line with the World Falls Guidelines (12), focusing on older adults referred for physiotherapy following a fall rather than all dizziness presentations. The DHT was incorporated into routine care with only minor adjustments to existing workflows. Only 10% of patients who fell due to intrinsic factors reported dizziness, a higher proportion may have experienced vestibular symptoms without recognising them, a phenomenon consistent with vestibular agnosia, where conscious awareness of vestibular impairment is reduced despite its presence (28). Postural hypotension was identified in ten patients and represents an important alternative or coexisting contributor to falls, and given the multifactorial nature of falls in older adults, vascular and vestibular mechanisms should not be considered mutually exclusive.

Despite the older age and high comorbidity burden of the feasibility cohort, 62% of patients were able to undergo DHT, demonstrating its practical feasibility in a busy ED environment. All eight patients who tested positive for BPPV received CRM, and none were readmitted within three months. While encouraging, this observation should be interpreted cautiously given the small sample size and lack of a comparator group, and it does not permit any definitive conclusions regarding treatment efficacy. Rather, the absence of readmissions raises the possibility that timely management may be beneficial, a hypothesis that warrants evaluation in a larger, controlled cohort. Environmental factors, particularly lack of space and limited trolley availability, were the most common barriers to completion and represent actionable targets for service improvement.

Consistent with our audit findings, onward referral rates in the feasibility cohort were low, with 53% receiving no documented referral. Without structured follow-up, it is unclear whether symptoms recurred or remained untreated in the community. A structured approach to acute vertigo management in ED has been advocated previously (4, 5, 17, 18, 24), and our findings support that targeted changes to AHP practice can be implemented with minimal operational impact. Implementing a vestibular pathway within ED has previously been associated with improved diagnostic accuracy and reductions in unnecessary imaging and inappropriate pharmacological management (22, 26, 29). Based on these findings, a DHT may be useful for a select group of patients in ED. A pragmatic starting point would be to offer adults aged 65 years and over who present to the ED following a fall a DHT as part of a structured assessment by a trained allied health professional. This approach should use simple pre-specified threshold criteria to guide selection rather than vestibular symptoms as an entry criterion.

### Limitations

This project was conducted in 2022, and practice may have evolved since data collection. Consequently, the findings should be interpreted within the context of the care pathways and clinical guidance available at that time.

The retrospective audit was limited by variable documentation quality, which affected the ability to complete several clinical fields, including in some cases the location of assessment. Additionally, the search strategy was designed to capture cases where BPPV was clinically considered rather than all dizziness presentations. Consequently, records coded only as “dizziness” or “unsteadiness” were excluded, which may have led to under ascertainment of BPPV cases. Therefore, the diagnostic management rates reported here likely reflect a “vestibular aware” subset of encounters and may not represent the broader acute dizziness population. Finally, although joint calibration was undertaken, some degree of inter-rater variability remains likely. As detailed in Supplementary Figure 1, cases with missing information or inaccessible records were excluded rather than imputed; a complete-case approach was used throughout.

Reasons for non-completion of DHT and CRM in the audit were not identifiable from the records reviewed. Omissions may reflect clinical judgement, lack of training, or environmental constraints, limiting interpretation of the low completion rates observed.

Neither length of stay nor time to diagnosis was captured in the retrospective audit. These are clinically meaningful outcomes, particularly in the context of fragmented care and multiple team involvement, and their absence limits the characterisation of acute management pathways. Future evaluations should prioritise collection of these metrics.

The feasibility study was deliberately limited to patients presenting following a fall, as expanding to all dizziness presentations was not achievable within existing clinical staffing. Environmental and patient-specific factors in the ED prevented assessment in some cases. Complete clinical data were available for 79 of 102 patients, the missing records arose from limitations in retrospective recovery of paper-based feasibility documentation and may have introduced selection bias if patients with incomplete records differed systematically from those included in the analysis. If patients with incomplete records were more likely to have had testing abandoned due to clinical complexity or environmental constraints, the completion rate of 62% reported here may represent an optimistic estimate of real-world feasibility, and should be interpreted accordingly. The time and resources required to train staff were not quantified, which limits assessment of the approach’s practicality and scalability in other acute settings. Notably, Bicknell et al. (30) reported that implementation of BPPV assessment and treatment was not feasible in inpatient areas; however, contextual differences in staffing and workflow may account for this divergence. Patient-reported experience was not formally captured, and future work should seek to incorporate this alongside clinical outcomes.

## Conclusion

Across both the retrospective audit and feasibility study, a consistent picture emerged of missed opportunity to assess and treat BPPV within acute care. Despite frequent neuroimaging, confirmed stroke was rare in patients presenting with isolated dizziness, whilst bedside assessment tools were used infrequently and vestibular suppressants overprescribed. The feasibility study demonstrated that DHT and CRM are achievable in real-world ED conditions, even in older adults with multiple comorbidities, and the absence of readmissions in this small, uncontrolled cohort is an encouraging finding warranting evaluation in a larger, controlled cohort. These findings highlight the need for standardised acute vertigo pathways, improved AHP integration, and targeted multi-professional training to support accurate diagnosis, reduce unnecessary investigations, and enhance the quality and consistency of vestibular care.

## Data Availability

The datasets presented in this article are not readily available because they contain information derived from clinical records that is subject to institutional and privacy protections. Access to a limited dataset may be considered upon reasonable request, subject to institutional approval, execution of an appropriate data-use agreement, and applicable privacy protections. Requests to access the datasets should be directed to the corresponding author.
