# Supplementary material for: Diagnostic and therapy gaps in BPPV care: a retrospective analysis and feasibility evaluation across a London NHS trust
Source: Front Neurol. 2026 Oct 14;17:1950987. doi: 10.3389/fneur.2026.1950987 (PMC13713311; doi:10.3389/fneur.2026.1950987)

Supplementary Figure 1. Case identification and data flow

Part 1: Retrospective Clinical Notes Evaluation

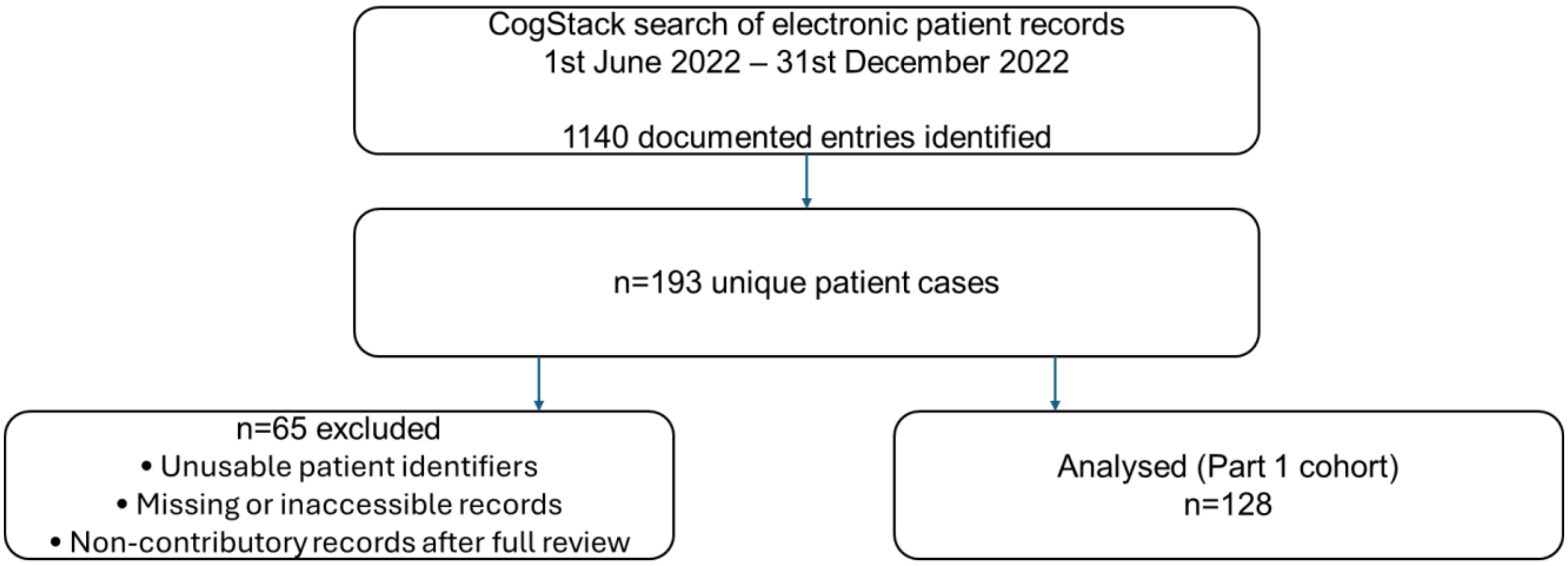

Part 2: Real-World Feasibility Implementation

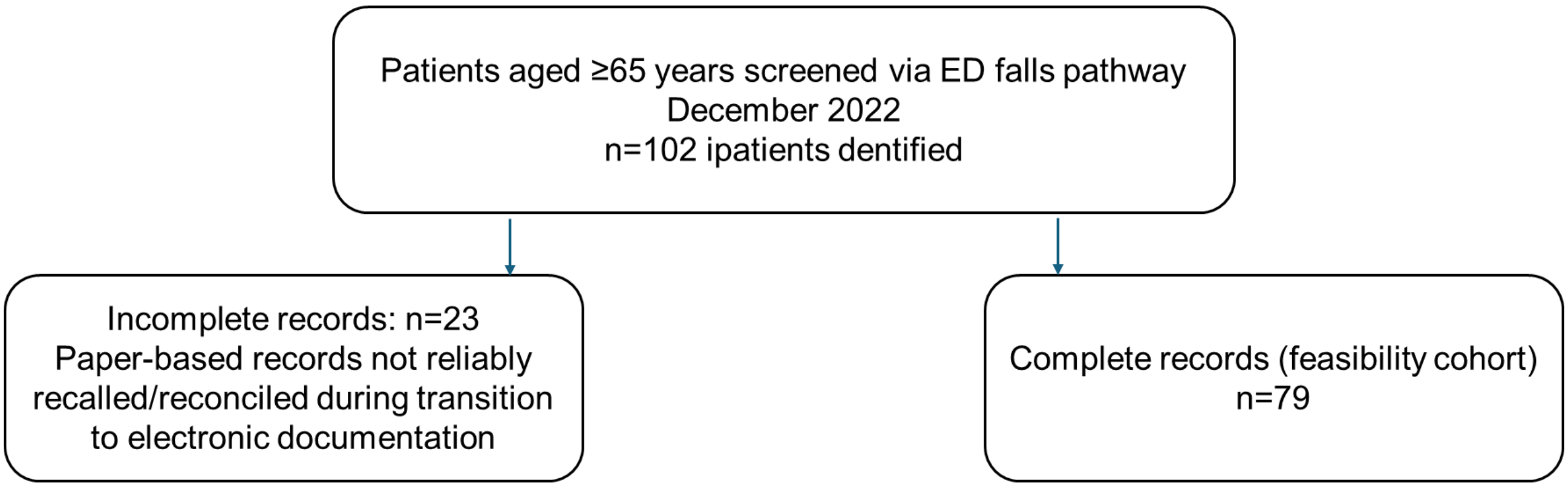

DHT screening outcomes reported on the full initial cohort (n=102), as testing was attempted before the completeness of subsequent documentation was known:

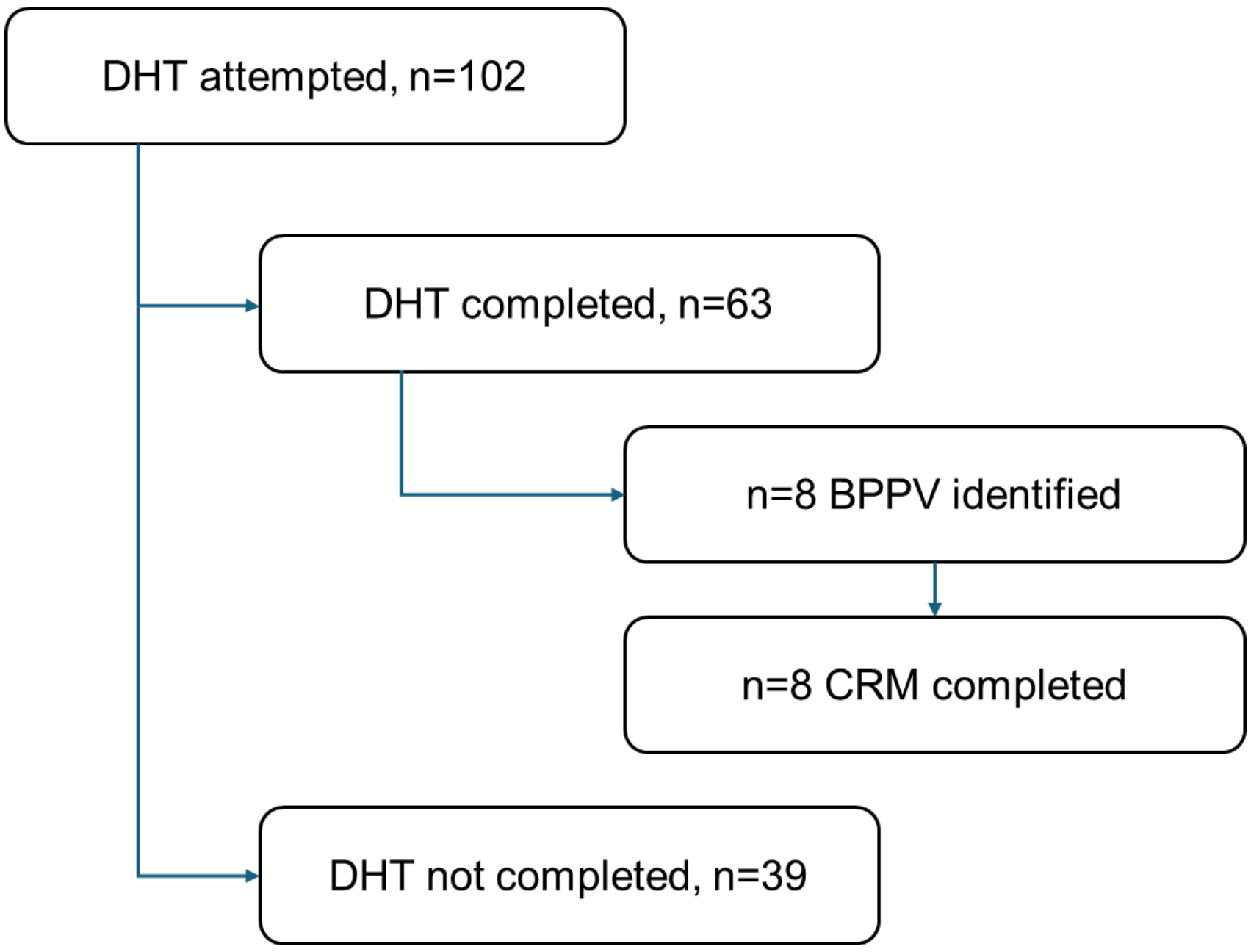

Supplement: Supplementary file 1 [file Data_Sheet_1.PDF]
