# Supplementary material for: Diagnostic and therapy gaps in BPPV care: a retrospective analysis and feasibility evaluation across a London NHS trust
Source: Front Neurol. 2026 Oct 14;17:1950987. doi: 10.3389/fneur.2026.1950987 (PMC13713311; doi:10.3389/fneur.2026.1950987)
Supplement: Supplementary file 2 [file Table_1.docx]

STROBE Statement—checklist of items that should be included in reports of observational studies

|  | Item No. | Recommendation | Section | Relevant text from manuscript |
| --- | --- | --- | --- | --- |
| **Title and abstract** | 1 | (*a*) Indicate the study’s design with a commonly used term in the title or the abstract | Title  Abstract: Methods | Title includes “Retrospective Analysis and Feasibility Evaluation Across a London NHS Trust”; abstract describes a retrospective review and a prospective feasibility study |
|  |  | (*b*) Provide in the abstract an informative and balanced summary of what was done and what was found | Abstract | Abstract provides structured summary of background, methods, results, and conclusions |
| Introduction | | | |  |
| Background/rationale | 2 | Explain the scientific background and rationale for the investigation being reported | -Introduction | Introduction describes prevalence of BPPV, variability in ED assessment and management, the role of AHPs, and rationale for the two-part evaluation |
| Objectives | 3 | State specific objectives, including any prespecified hypotheses | -Introduction  -Materials and Methods: Statistical analysis | Two objectives stated. Part 1: characterise assessment and management of BPPV and identify gaps in practice. Part 2: evaluate feasibility of AHP-led DHT screening in the ED falls pathway. Secondary objectives: diagnostic yield, treatment delivery, implementation barriers, short-term dizziness-related reattendance  No formal hypothesis testing undertaken |
| Methods | | | |  |
| Study design | 4 | Present key elements of study design early in the paper | -Abstract: Methods  -Materials and methods: Study design | Two linked components: retrospective review of electronic patient records (Part 1) and prospective feasibility evaluation (Part 2); completed STROBE checklist provided as Supplementary Material  Part 1 drew on both Trust sites (aggregate data); Part 2 was conducted at a single ED site |
| Setting | 5 | Describe the setting, locations, and relevant dates, including periods of recruitment, exposure, follow-up, and data collection | -Materials and Methods: Part 1, Part 2 | Part 1: two hospital sites within a London NHS Trust; consecutive seven-month period (1 June to 31 December 2022)  Part 2: one ED site; one month (1 to 31 December 2022) |
| Participants | 6 | (*a*) *Cohort study*—Give the eligibility criteria, and the sources and methods of selection of participants. Describe methods of follow-up  *Case-control study*—Give the eligibility criteria, and the sources and methods of case ascertainment and control selection. Give the rationale for the choice of cases and controls  *Cross-sectional study*—Give the eligibility criteria, and the sources and methods of selection of participants | -Materials and Methods: Part 1, Part 2 | Part 1: records identified by CogStack search using predefined terms selected by consensus; duplicate, repeat, and non-contributory entries excluded by trained clinicians  Part 2: patients aged ≥65 years receiving routine falls assessments in the ED |
|  |  | (*b*) *Cohort study*—For matched studies, give matching criteria and number of exposed and unexposed  *Case-control study*—For matched studies, give matching criteria and the number of controls per case | N/A | N/A |
| Variables | 7 | Clearly define all outcomes, exposures, predictors, potential confounders, and effect modifiers. Give diagnostic criteria, if applicable | -Materials and Methods: Part 1, Part 2 | Part 1: diagnosis, imaging, medication, vestibular assessment, and re-admission extracted using a predefined proforma with operational definitions (Supplementary Material)  Part 2: positive BPPV assessments, non-completion with limiting factors, and implementation fidelity; falls classified as intrinsic or extrinsic |
| Data sources/ measurement | 8* | For each variable of interest, give sources of data and details of methods of assessment (measurement). Describe comparability of assessment methods if there is more than one group | -Materials and Methods: Part 1, Part 2 | Part 1: electronic patient records searched via CogStack; data extracted by five therapists using a structured proforma  Part 2: physiotherapy documentation; DHT and nystagmus assessed by direct clinical observation, without video-oculography |
| Bias | 9 | Describe any efforts to address potential sources of bias | -Materials and Methods: Part 1  -Limitations | Bias addressed through predefined search terms selected by consensus, structured proforma with operational definitions, five independent reviewers, and an initial calibration exercise on sample cases (a subset of five cases jointly reviewed); discrepancies and ambiguous cases resolved by consensus. Residual bias discussed in Limitations |
| Study size | 10 | Explain how the study size was arrived at | -Materials and Methods: Part 1, Part 2 | Study includes all eligible cases identified during defined study periods (Part 1: seven months; Part 2: one month) |

Continued on next page

| Quantitative variables | 11 | Explain how quantitative variables were handled in the analyses. If applicable, describe which groupings were chosen and why | -Materials and Methods: Statistical analysis  Results | Continuous variables reported as mean (SD); categorical variables as frequencies (%); 95% CIs (Wilson method) for selected key proportions |
| --- | --- | --- | --- | --- |
| Statistical methods | 12 | (*a*) Describe all statistical methods, including those used to control for confounding | -Materials and Methods: Statistical analysis | Descriptive analysis in Microsoft Excel; 95% CIs (Wilson method) for selected key proportions; no formal hypothesis testing or between-group inferential analyses |
|  |  | (*b*) Describe any methods used to examine subgroups and interactions | N/A | N/A |
|  |  | (*c*) Explain how missing data were addressed | -Results: Part 1, Part 2  -Limitations | Complete-case approach used throughout; no imputation. Cases with missing information or inaccessible records excluded |
|  |  | (*d*) *Cohort study*—If applicable, explain how loss to follow-up was addressed  *Case-control study*—If applicable, explain how matching of cases and controls was addressed  *Cross-sectional study*—If applicable, describe analytical methods taking account of sampling strategy | -Results: Part 1, Part 2 | Limited applicability. Three-month re-admission used as follow-up measure |
|  |  | (*e*) Describe any sensitivity analyses | N/A | N/A |
| Results | | | | |
| Participants | 13* | (a) Report numbers of individuals at each stage of study—eg numbers potentially eligible, examined for eligibility, confirmed eligible, included in the study, completing follow-up, and analysed | -Results: Part 1, Part 2 | Part 1: 1,140 document entries identified; 193 unique patient cases; 128 included  Part 2: 102 patients seen; complete records for 79 (participant characteristics) and 102 for selected implementation outcomes |
|  |  | (b) Give reasons for non-participation at each stage | -Results: Part 1, Part 2 | Part 1: duplicate and non-contributory records removed  Part 2: records that could not be reliably recovered (paper-based documentation); reasons for DHT non-completion in Table 3 |
|  |  | (c) Consider use of a flow diagram | -Results: Part 1, Part 2 | Flow diagram for Part 1 and Part 2 provided as Supplementary Figure 1 |
| Descriptive data | 14* | (a) Give characteristics of study participants (eg demographic, clinical, social) and information on exposures and potential confounders | -Results: Part 1, Part 2 | Participant characteristics presented in Table 1 (Part 1, n=128) and Table 2 (Part 2, n=79) |
|  |  | (b) Indicate number of participants with missing data for each variable of interest | -Results: Part 1, Part 2  -Limitations | Missing data described for feasibility cohort (79/102 analysed); Table 2 footnote identifies which rows use each denominator. Part 1 documentation gaps described in Limitations |
|  |  | (c) *Cohort study*—Summarise follow-up time (eg, average and total amount) | -Results: Part 1, Part 2 | Follow-up period of three months for re-admission outcomes only |
| Outcome data | 15* | *Cohort study*—Report numbers of outcome events or summary measures over time | -Results: Part 1, Part 2 | Outcome data reported as numbers and proportions (95% CIs for selected key proportions) for diagnosis, assessment, treatment, and re-admission |
|  |  | *Case-control study—*Report numbers in each exposure category, or summary measures of exposure | N/A | N/A |
|  |  | *Cross-sectional study—*Report numbers of outcome events or summary measures | N/A | N/A |
| Main results | 16 | (*a*) Give unadjusted estimates and, if applicable, confounder-adjusted estimates and their precision (eg, 95% confidence interval). Make clear which confounders were adjusted for and why they were included | -Results: Part 1, Part 2 | Main findings reported as descriptive proportions with 95% CIs (Wilson method) for selected key outcomes; no confounder adjusted estimates |
|  |  | (*b*) Report category boundaries when continuous variables were categorized | N/A | N/A |
|  |  | (*c*) If relevant, consider translating estimates of relative risk into absolute risk for a meaningful time period | N/A | N/A |

Continued on next page

| Other analyses | 17 | Report other analyses done—eg analyses of subgroups and interactions, and sensitivity analyses | N/A | N/A |
| --- | --- | --- | --- | --- |
| Discussion | | | | |
| Key results | 18 | Summarise key results with reference to study objectives | -Discussion  -Conclusion | Key findings summarised: low rates of assessment and treatment, frequent neuroimaging, vestibular suppressant prescribing, and feasibility of DHT (completed in 62%); absence of readmissions in the feasibility cohort interpreted cautiously |
| Limitations | 19 | Discuss limitations of the study, taking into account sources of potential bias or imprecision. Discuss both direction and magnitude of any potential bias | -Limitations | Limitations include data from 2022, variable documentation quality, under-ascertainment from the search strategy, inter-rater variability, complete-case exclusion and possible selection bias (completion rate may be optimistic), reasons for non-completion not identifiable, length of stay and time to diagnosis not captured, training resources not quantified, and patient experience not captured |
| Interpretation | 20 | Give a cautious overall interpretation of results considering objectives, limitations, multiplicity of analyses, results from similar studies, and other relevant evidence | -Discussion  -Conclusion | Interpretation contextualised with prior literature; NICE NG249 (2025) cited as current best-practice framework rather than the standard in 2022; Part 2 treated as feasibility level data from a distinct population; absence of readmissions not taken as evidence of efficacy |
| Generalisability | 21 | Discuss the generalisability (external validity) of the study results | -Discussion  -Limitations | Part 1 reflects a “vestibular aware” subset and may not represent the broader acute dizziness population; Part 2 was at a single ED site in patients following a fall; findings interpreted within 2022 pathways and guidance; staffing and workflow differences may limit transferability |
| Other information | |  | | |
| Funding | 22 | Give the source of funding and the role of the funders for the present study and, if applicable, for the original study on which the present article is based | -Funding | No financial support received for the research, authorship, and/or publication of this article |

*Give information separately for cases and controls in case-control studies and, if applicable, for exposed and unexposed groups in cohort and cross-sectional studies.

**Note:** An Explanation and Elaboration article discusses each checklist item and gives methodological background and published examples of transparent reporting. The STROBE checklist is best used in conjunction with this article (freely available on the Web sites of PLoS Medicine at http://www.plosmedicine.org/, Annals of Internal Medicine at http://www.annals.org/, and Epidemiology at http://www.epidem.com/). Information on the STROBE Initiative is available at www.strobe-statement.org.
